# Galectin-1 inhibits oral-intestinal allergy syndrome

**DOI:** 10.18632/oncotarget.14571

**Published:** 2017-01-10

**Authors:** Rui-Di Xie, Ling-Zhi Xu, Li-Tao Yang, Shuai Wang, Qi Liu, Zhi-Gang Liu, Ping-Chang Yang

**Affiliations:** ^1^ The Research Center of Allergy & Immunology, Shenzhen University School of Medicine, Shenzhen, China; ^2^ Periodontal Department, the Affiliated Hospital of Zunyi Medical College, Zunyi, China; ^3^ The ENT Hospital of Shenzhen University and Shenzhen ENT Institute, Shenzhen, China; ^4^ Brain Body Institute, McMaster University, Hamilton, ON, Canada

**Keywords:** oral mucosa, oral allergy, peanut, micro RNA-98, galectin-1

## Abstract

**Background and aims:**

The pathogenesis of oral-intestinal allergy syndrome (OIAS) has not been well understood. Published data indicate that galectin (Gal) 1 has immune regulatory functions. This study tests a hypothesis that Gal1 inhibits oral-intestinal allergy syndrome.

**Methods:**

Mice were sensitized to peanut extracts (PE) via the buccal mucosa with or without using Gal1 together.

**Results:**

Upon re-exposure to specific antigen, the OIAS mice showed the systemic allergic response, the oral allergic reactions, and intestinal allergic inflammation, including increases in serum histamine, drop of the core temperature, higher levels of PE-specific IgE and interleukin (IL)-4. Increases in mast cell and eosinophil in the oral mucosa and intestinal mucosa were also observed. The OIAS was inhibited by co-administration with Gal1 via a mechanism of suppressing micro RNA (miR)-98 and reversing the expression of IL-10 in CD14+ cells in the intestine.

**Conclusions:**

The OIAS can be induced by applying specific antigens to the oral mucosa, which can be inhibited by co-administration with Gal1.

## INTRODUCTION

The oral allergy syndrome is a common phenomenon. It belongs to the contact allergic response that occurs upon contacting specific antigens. The syndrome includes itching and swelling of the lips, palate and tongue, usually after eating the offending foods [1]. Thus, oral allergy syndrome is also considered a form of food allergy [2]. In some cases, oral allergic responses occur together with throat allergy and called the oral allergy syndrome [3]. Some patients with oral allergy are also allergic to pollen and have hay fever, implicating some antigens in foods and fruits is structurally similar to pollen [4]. One of the clinical features of oral allergy syndrome is the local mucosal edema. In the rare cases, the edema in the throat may cause difficult breathing, or even asphyxiation. Although the research about allergy has advanced rapidly in the recent years, the pathogenesis of allergic disorders is still obscure.

Interleukin (IL)-10 is an important immune regulatory cytokine. One of the functions of IL-10 is to suppress the inflammatory cytokine production [5]. IL-10 can be produced by a number of immune cells, including regulatory T cells, regulatory B cells, T helper (Th) 2 cells and monocytes [6, 7]. Monocytes can differentiate into dendritic cells or macrophages and play important role in immunity. Published data indicate that a fraction of monocyte expresses IL-10 that possesses immune regulatory functions [5]. Whether are IL-10-expression monocytes dysfunction in the oral allergy syndrome is to be investigated. It is reported that micro RNA (miR)-98 suppresses the IL-10 expression in macrophages [8]. miRs are single strand non-coding RNA chain with 18-22 nucleotides in length. The miRs regulate target gene expression post transcriptionally. Whether IL-10-expression monocytes dysfunction plays a role in the oral allergy syndrome is to be investigated.

Published data indicate that galectin-1 (Gal1) is involved in the regulation of immune functions [9]. Whether Gal1 can alleviate oral allergy has not been investigated. Since some foods can be the offending antigens of oral allergy [10], we hypothesize that oral allergy and intestinal allergy may concomitantly occur, a phenomenon may be designated the “oral-intestinal allergy syndrome (OIAS)”. To test this, we sensitized mice by application of antigens to the mouse buccal mucosa. The mice showed allergic responses in the oral mucosa as well as intestinal mucosa. The OIAS symptoms were inhibited by Gal1.

## RESULTS

### Induction of oral-intestinal allergic response (OIAS) by exposing oral mucosa to antigens

**Figure 1:**
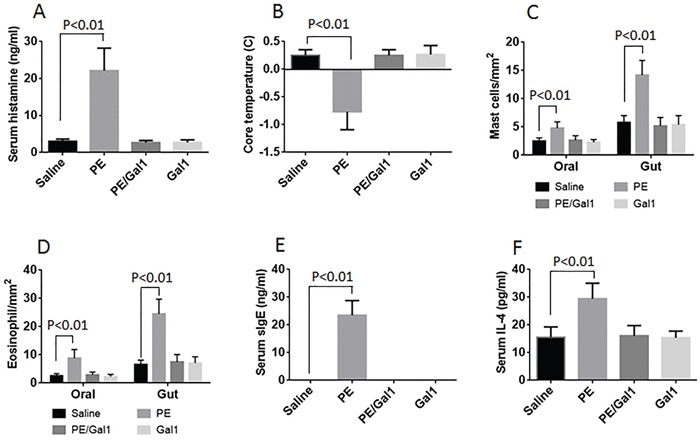
Allergic response in allergic mice The bars indicate the serum histamine **A**., drop of the core temperature **B**., counts of mast cell/eosinophil in the oral and intestinal mucosa **C-D**., serum specific IgE (sIgE) **E**. and serum IL-4 **F**. in mice. The treatment of mice is denoted on the X axis. Each dot represents an individual datum. Saline: Mice were treated with saline used as controls. PE: Mice were sensitized to PE. Gal1: Mice received Gal1 via intraperitoneal injection. Each group consists of 6 mice. The data were summarized from 6 independent experiments.

Mice were sensitized with PE via contacting the oral mucosa with PE and CT. After re-exposure to the specific antigen PE, the mice were sacrificed; the allergic responses were assessed. The results showed that high infiltration of eosinophils and mast cells (Supplementary Figure 1 and Supplementary Figure 2 in supplemental materials) in the oral mucosa and intestinal mucosa. The PE-specific IgE was detected in the sera. Compared to control mice, the sensitized mice showed higher levels of serum histamine and IL-4. In addition, 30 min after exposure to the PE, the drop of core temperature and diarrhea were observed in the sensitized mice (Figure 1). The data indicate that sensitizing the oral mucosa can induce allergic responses in both oral mucosa and intestinal mucosa; this phenomenon can be designated OIAS.

### Gal1 inhibits OIAS

During the sensitization period, mice were treated with or without Gal1. The results showed that administration of Gal1 inhibited OIAS (Figure 1).

### Gal1 promotes development of IL-10+ CD14+ cells in the immunized mice

**Figure 2:**
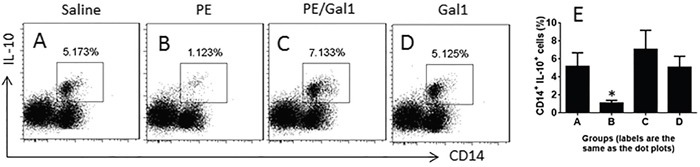
Gal1 facilitates the generation of IL-10+ CD14+ cells **A-D**. the gated dot plots indicate the frequency of IL-10+ CD14+ cells in the mouse intestine. The treatment of mice is denoted above each panel. **E**. the summarized data of (A-D). Saline: Mice were treated with saline used as controls. PE: Mice were sensitized to PE. Gal1: Mice received Gal1 via intraperitoneal injection. Each group consists of 6 mice. The data represent 6 independent experiments.

Prompted by published data that IL-10-producing CD14+ cells (IL-10+ CD14+ cells) are an important fraction in the immune regulation [11], we hypothesized that the immunization of mice with antigen PE might affect IL-10+ CD14+ cells. Thus, we assessed IL-10+ CD14+ cells in the LPMCs of allergic mice. The results showed that the frequency of IL-10+ CD14+ cells was about 5.17% in LPMCs of control mice, which was only 1.12% in allergic mice. In the mice treated with both PE and Gal1, however, the frequency of IL-10+ CD14+ cells in LPMCs was about 7.13%. The results indicate that Gal1 promoted the development of IL-10+ CD14+ cells in the mouse intestine (Figure 2).

### CD14+ cells from allergic mice express higher levels of miR-98 and lower levels of IL-10

**Figure 3:**
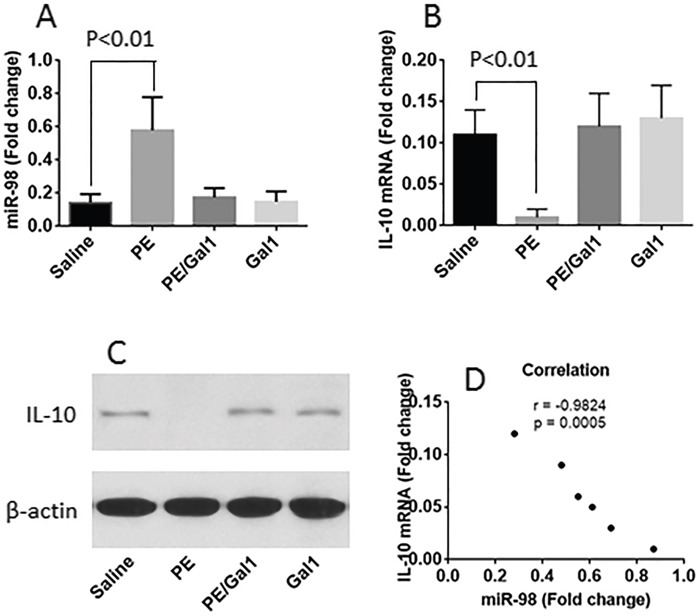
miR-98 and IL-10 levels in CD14+ cells The levels of miR-98 **A**. IL-10 mRNA **B**. and IL-10 protein **C**. in CD14+ cells; the cells were isolated from LPMCs of mice treated with the reagents denoted on the X axis. **D**., the negative correlation between IL-10 mRNA and miR-98 in CD14+ cells of the allergic mouse intestine. Each group consists of 6 mice.

Published data indicate that miR-98 suppresses the expression of IL-10 [8]. Since the frequency of IL-10+ B cells was lower in CD14+ cells of allergic mice as shown by Figure 2, we inferred that the expression of miR-98 might be higher in the CD14+ cells of allergic mice. To test this, we isolated CD14+ cells from LPMCs and analyzed by RT-qPCR and Western blotting. The results showed that the levels of miR-98 were detectable in CD14+ cells of naive control mice, which was significantly enhanced in allergic mice. Treating mice with both Gal1 and PE, or Gal1 alone did not increase the expression of miR-98 in CD14+ cells (Figure 3A). On the other hand, the IL-10 levels in the CD14+ cells were markedly lower in the sensitized mice at both mRNA levels and protein levels (Figure 3B–3C). A correlation test was performed with the data of miR-98 and IL-10 mRNA in CD14+ cells. The results showed a negative correlation between miR-98 and IL-10 expression in CD14+ cells (Figure 3D), suggesting miR-98 might interfere with the expression of IL-10.

### IL-4 increases miR-98 expression in CD14+ cells

**Figure 4:**
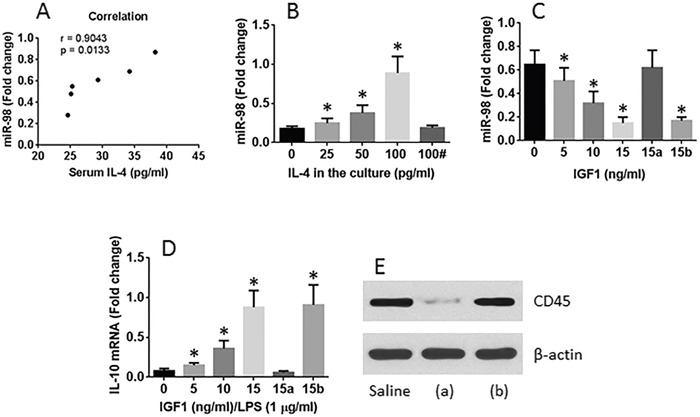
Gal1 suppresses miR-98 and reverses IL-10 expression in CD14+ cells **A**. positive correlation between serum IL-4 levels and miR-98 in intestinal CD14+ cells of allergic mice. **B**. the levels of miR-98 in CD14+ cells. The CD14+ cells were isolated from the naive mouse spleen after exposure to IL-4 in the culture for 48 h. #, Gal1 in the culture (15 ng/ml). **C**. the levels of miR-98 in CD14+ cells isolated from the OIAS mouse intestine after exposure to Gal1 in the culture for 48 h. **D**. the levels of IL-10 mRNA in CD14+ cells isolated from the allergic mouse intestine after exposure to Gal1 and LPS in the culture for 48 h. **E**. the CD45 RNAi results; (a) CD14+ cells were treated with CD45 RNAi; (b) CD14+ cells were treated with control RNAi. *, p <0.01, compared with the 0 group. The data were summarized from 3 independent experiments.

We then performed a correlation test with the data of serum IL-4 and the miR-98 levels in CD14+ cells isolated from LPMCs of allergic mice; the results showed a positive correlation (Figure 4A). The data implicate that IL-4 may enhance the expression of miR-98 in CD14+ cells. To test this, we isolated CD14+ cells from naive mouse intestine and stimulated with IL-4 in the culture for 48 h. The results showed that IL-4 did enhance the expression of miR-98 in CD14+ cells in an IL-4 concentration-dependent manner (Figure 4B).

### Gal1 suppresses miR-98 in CD14+ cells

We then tested the role of Gal1 in the regulation of miR-98 expression in CD14+ cells. CD14+ cells were isolated from the LPMCs of allergic mice. The cells expressed high levels of miR-98. The allergy mouse-derived CD14+ cells were treated with Gal1 in the culture for 48 h. Indeed, Gal1 inhibited the levels of miR-98 in CD14+ cells (Figure 4C).

### Gal1 reverses the ability of IL-10 expression in CD14+ cells

We then tested if Gal1 reversed the ability of IL-10 expression in allergy mouse-derived CD14+ cells. The cells were exposed to Gal1 and LPS (a stimulator of IL-10 expression) in the culture for 48 h. The results showed that the expression of IL-10 was increased in an Gal1 dose-dependent manner (Figure 4D).

### CD45 mediates the effects of Gal1 on modulating the expression of IL-10 and miR-98 in CD14+ cells

Since CD45 is the receptor of Gal1 [12], we next assessed if CD45 mediates the effects of Gal1 in the regulation of the expression of IL-10 and miR-98 in CD14+ cells. Firstly we prepared the CD45-deficient CD14+ cells by transducing the CD45 shRNA-carrying lentivirus or non-specific shRNA-carrying virus (Figure 4E). CD14+ cells were processed as described in Figure 4C and Figure 4D. The results showed that the CD45-deficient CD14+ cells did not respond the stimuli of Gal1 (Figure 4C–4D), indicating that Gal1 modulates the expression of miR-98 and IL-10 in CD14+ cells via ligating CD45.

### IL-10+ CD14+ cells inhibit oral-intestinal syndrome

**Figure 5:**
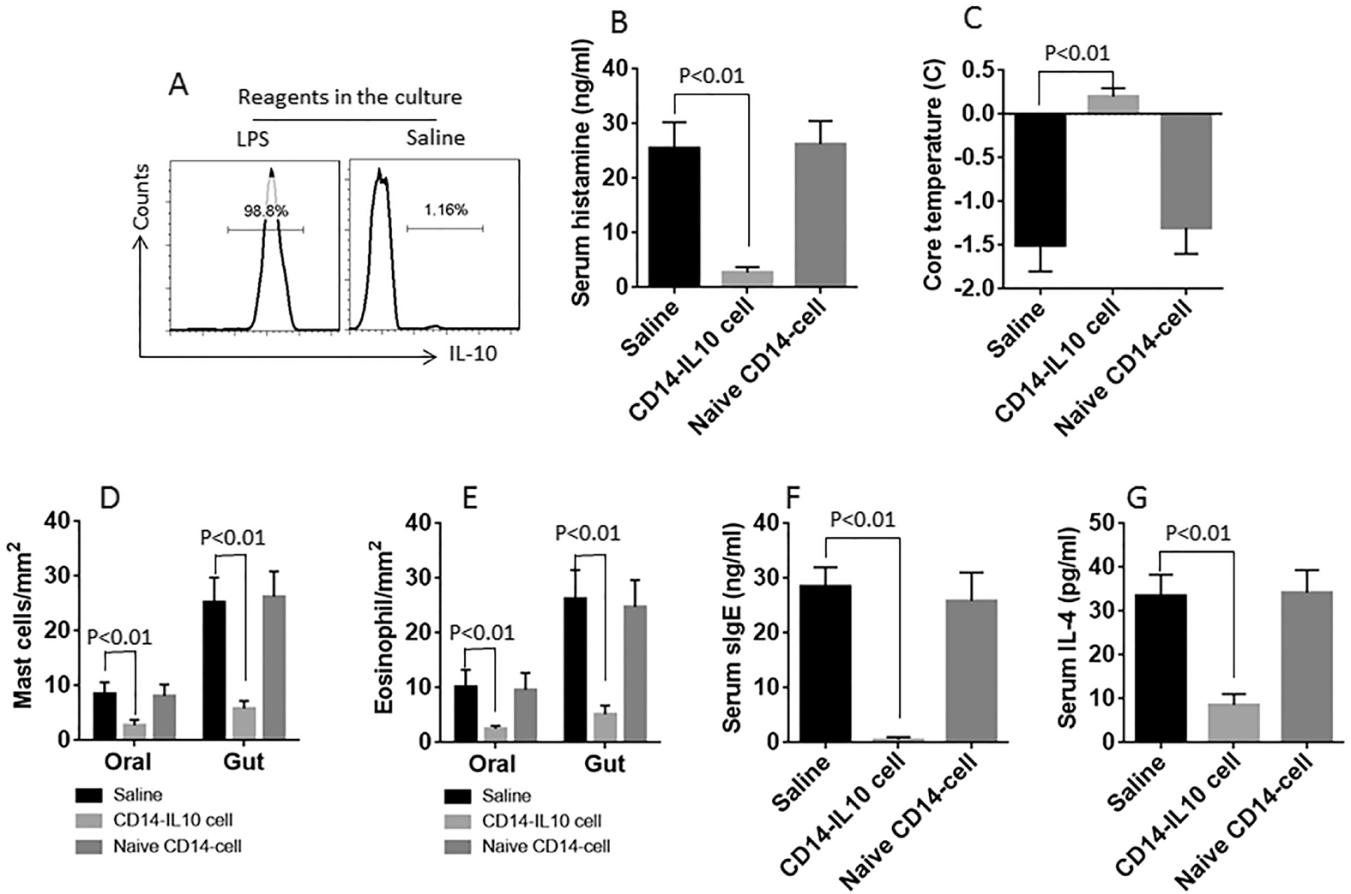
IL-10+ CD14+ cells inhibit oral-intestinal syndrome **A**. the gated flow cytometry histograms show the frequency of IL-10+ CD14+ cells after treating with the reagents denoted above each histogram. **B-G**. the bars show the results of serum histamine (B), changes of the core temperature (C), counts of mast cell (D) and eosinophil (E) in the buccal mucosa and the intestinal mucosa, serum specific IgE (F) and serum IL-4 (G) of mice (with the oral-intestinal syndrome) after the treatment denoted on the X axis. Each group consists of 6 mice. Data of the bars were summarized from 6 independent experiments.

Data reported above implicate the IL-10+ CD14+ cells may have immune regulatory functions on the oral-intestinal syndrome. To test this, we generated IL-10+ CD14+ cells to be adoptively transferred into mice during the period of sensitization. Indeed, the oral-intestinal allergy syndrome was abolished by the IL-10+ CD14+ cells (Figure 5; Supplementary Figure 1; Supplementary Figure 2).

## DISCUSSION

The present data show the oral-intestinal allergy syndrome (OIAS) in mice. By immunizing mice via applying antigens to the buccal mucosa and re-challenging mice with specific antigens, mice showed systemic allergic responses and allergic responses in the buccal mucosa and intestinal mucosa. Lower frequency of IL-10+ CD14+ cells was detected in mice with the OIAS. CD14+ cells from the intestinal mucosa of OIAS mice showed lower levels of IL-10 expression and higher levels of miR-98; the latter could be up regulated by exposure to IL-4 in the culture. Administration with Gal1 suppressed the expression of miR-98 in CD14+ cells and inhibited the OIAS in mice. The allergic response in the oral mucosa of the OIAS mice is that heavy infiltration of mast cells and eosinophils were observed in the oral mucosa.

The oral allergy syndrome was reported a long time ago [1]. Because it can occur together with hay fever, a link between the oral allergy syndrome and pollen sensitization was proposed [13]. The present data revealed another aspect of the oral allergy syndrome that also can occur together with intestinal allergy.

The data also showed that lower levels of IL-10 expression in the intestine-isolated CD14+ cells. IL-10 is a cytokine possesses important immune regulatory functions. The IL-10 deficiency has been reported in a number of immune disorders. Such as asthma patients showed lower serum levels of IL-10, which positively correlated with the lung function [14]. The serum IL-10 levels in atopic eczema/dermatitis syndrome children were markedly lower than that in healthy controls [15]. Our data are in line with those previous reports by showing the frequency of IL-10+ CD14+ cell was lower in OIAS mice than that in control mice.

The mechanism underlying the suppression of IL-10 is not clear. A recent report indicated that miR-98 suppressed the expression of IL-10 in macrophages. The authors found that miR-98 targeted the 3′untranslated region of the IL-10 transcript. Overexpression of miR-98 inhibited LPS-induced IL-10 expression [8]. The present data demonstrate that miR-98 expression was enhanced in CD14+ cells of OIAS mice and negatively correlated with the expression of IL-10.

The data show that Gal1 can inhibit the PE-induced OIAS. The underlying mechanism may be because Gal1 has multiple biological functions. It is known that Gal1 has the immune suppression functions [9]. By promoting the secretion of thymic stromal lymphopoietin, epidermal growth factor, IL-10, IL-25, and transforming growth factor-β1 by intestinal epithelial cells, Gal1 can inhibit experimental intestinal inflammation [16]. It induces IL-10 expression in dendritic cells and CD4+ T cells to facilitate the generation of immune regulatory cells [17]. Gal1 is also involved in maintaining the homeostasis in the eye [18]. Our results have expanded the existing knowledge in the study of Gal1 by showing that Gal1 can inhibit the OIAS in mice.

The data show that the frequency of IL-10+ CD14+ cell in the intestine was less in the OIAS mice as compared to naïve mice. Our previous work also found that IL-10-producing B cells were less in the intestine of mice with food allergy [19, 20]. Clinical study indicates that IL-10 gene polymorphism is associated with food allergy and lower serum IL-10 levels [21]. Others also found that the myeloid-derived monocytes express IL-10, which suppresses most immune effector cells [22]. Because IL-10 is an anti-inflammation cytokine; it suppresses allergic responses [23], in which IL-10 inhibits the expression of the high affinity IgE receptor via the signal transducer and activator of transcription (STAT)-3. IL-10 also diminishes IgE-induced response by inhibiting expression of the signaling molecules Syk, Fyn, Akt, and STAT5 [24]. the finding of decrease in IL-10+ CD14+ cells in the intestine may be responsible, at least in part, in the pathogenesis of OIAS. The reasoning is supported by the subsequent data. By adoptive transfer with IL-10+ CD14+ cells, the symptoms of OIAS were efficiently inhibited in the mice as shown by the present data. Other also found that IL-10-producing B cells down regulated inflammation in airway hyper-responsiveness [25].

'In summary, the present data show that immunization at the oral mucosa induced OIAS in mice via suppressing IL-10 expression in CD14+ cells in the intestine.

## MATERIALS AND METHODS

### Mice and ethic statement

Male BALB/c mice were purchased from the Guangdong Experimental Animal Center. The mice were maintained in a pathogen-free environment with accessing food and water freely. The experimental procedures were approved by the Animal Ethics Committee at Shenzhen University. The experiments were performed in accordance with the approved guidelines.

### Preparation of peanut antigens

Peanut proteins were extracted from crude peanut according to previously reported procedures [26, 27]. The peanut extracts (PE) were used as a specific antigen in the present study.

### Sensitization of mice and treatment with Gal1

A sensitization mixture was made of PE and cholera toxin at a ratio of 10:1 (W/W), or PE/Gal1 and cholera toxin at a ratio of 10:10:1 (W/W/W). Mice were under light anesthesia by inspiration of ethyl; the sensitization mixture (soaked in a cotton ball), or Gal1 alone, was applied to the buccal mucosa of both sides, three times a day for 14 consecutive days. Caution was taken to avoid the antigen swallowing down the digestive tract by optimizing the amounts of the sensitization mixture in the cotton ball.

### Challenging mice with PE and sample collection

On day 15, the mice were challenged with PE (5 mg/mouse) in 0.3 ml saline via gavage-feeding as well as applied to the buccal mucosa. The core temperature was recorded from each mouse 30 min after the challenge. The mice were sacrificed 4 h later. The blood samples, buccal mucosa and small intestine were collected for further experiments.

### Assessment of serum levels of histamine and PE-specific IgE

The blood samples were collected from each mouse at the sacrifice. The sera were isolated from the blood by centrifugation and stored at -80 °C until use. The histamine levels were determined by enzyme-linked immunosorbent assay (ELISA) with a reagent kit (Biocompare) following the manufacturer's instructions. The serum levels of PE-specific IgE were assessed by an in-house ELISA with the reagents purchased from Abcam. Briefly, the 96-well microplates were coated with PE (20 μg/ml) in carbonate buffer (0.05 M, pH 9.6) overnight at 4 °C. The plates were blocked by incubating with 5% skim milk for 30 min at 37 °C. The serum samples (diluted 10 folds) or isotype IgG was added to the wells (0.1 ml/well; in triplicate) and incubated overnight at 4 °C; followed by adding anti-IgE antibodies or isotype IgG and incubated overnight at 4 °C; the second antibodies (labeled with peroxidase) were added and incubated at room temperature for 2 h. Washing with PBS was performed after each time of incubation. The plates were developed with Tetramethylbenzidine (TMB) substrate (Sigma Aldrich) following the manufacturer's instructions. The reaction was stopped by adding 2M H_2_SO_4_. The plates were read in a microplate reader (BioTek, Shanghai, China) at 450 nm. The readouts from wells of isotype IgG were regarded background and were subtracted from each sample well.

### Mast cell and eosinophil counts in the oral mucosa and intestinal mucosa

Immediately after the sacrifice, the buccal mucosa and a segment of the jejunum were excised and fixed with 4% formalin overnight. The tissue was processed for paraffin sections. The sections were stained with hematoxylin and eosin, or 0.5% toluidine blue. Mast cells and eosinophils in the sections were counted under a light microscope; 20 randomly selected fields were counted for each mouse. All the slides were coded. The observers were not aware of the code to avoid the observer bias.

### Assessment of serum IL-4 levels by ELISA

The serum levels of IL-4 were determined by ELISA with a reagent kit (R&D Systems) following the manufacturer's instructions.

### Flow cytometry

For the surface staining, cells were stained with fluorochrome-labeled antibodies or isotype IgG for 30 min at 4 °C. If the intracellular staining was required, the cells were fixed with 1% paraformaldehyde for 1 h, and permeated by incubation with 0.5% saponin for 30 min; the cells were then incubated with fluorochrome-labeled antibodies or isotype IgG for 30 min at 4 °C. After washing with PBS, the cells were analyzed with a flow cytometer (FACSCanto II, BD Bioscience). The data were analyzed by software flowjo. Data from the isotype IgG staining were used as a gating reference.

### Real time quantitative RT-PCR (RT-qPCR)

The levels of IL-10 mRNA and miR-98 were assessed by RT-qPCR. Total RNA was extracted from cells with the TRIzol reagents (Invitrogen). The cDNA was synthesized with the RNA and a reverse transcription kit (Invitrogen). PCR was performed in a real time PCR device (MiniOpticon, Bio-Rad) with the SYBR Green Master Mix (Invitrogen). The results were normalized to fold change against the internal control gene β-actin. The primers of IL-10 and miR-98 were provided by the Enke Biotech (Shenzhen, China).

### Western blotting

The total proteins were extracted from cells, separated by sodium dodecyl sulfate polyacrylamide gel electrophoresis (SDS-PAGE) and transferred onto a polyvinylidene difluoride (PVDF) membrane. After blocking with 5% skim milk at room temperature for 30 min, the membrane was stained with the primary antibodies or isotype IgG overnight at 4 °C, washed with PBS, followed by incubation with peroxidase-labeled second antibodies for 1 h at room temperature, washed with PBS again. The blots on the membrane were developed with enhanced chemiluminescence and photographed. The antibodies used in Western blotting were purchased from Santa Cruz Biotech (Santa Cruz, CA).

### RNA interference (RNAi)

CD14+ cells were isolated from LPMC by magnetic cells sorting (MACS) with specific reagent kit purchased from Miltenyi Biotech (San Diego, CA) following the manufacturer's instructions. The CD14+ cells were transduced with the CD45 shRNA-carrying lentivirus or non-specific shRNA-carrying lentivirus (Santa Cruz Biotech; Santa Cruz, CA) following the manufacturer's instructions. The effects of the RNAi were assessed by Western blotting.

### Statistical analysis

Data are presented as the mean ± standard deviation from at least three independent experiments. The difference between groups was analyzed by Student's t-test (unpaired t-test, two-tailed) or by two-way ANOVA followed by Bonferroni correction. The correlation test was performed with software Prism GraphPad. p <0.05 was considered significant.

## SUPPLEMENTARY FIGURES


