# Supplementary material for: Galectin-1 inhibits oral-intestinal allergy syndrome
Source: Oncotarget. 2017 Jan 10;8(8):13214–22. doi: 10.18632/oncotarget.14571 (PMC5355090; doi:10.18632/oncotarget.14571)
Supplement: Supplementary file 1 [file oncotarget-08-13214-s001.pdf]

Galectin-1 inhibits oral-intestinal allergy syndrome

SUPPLEMENTARY FIGURES

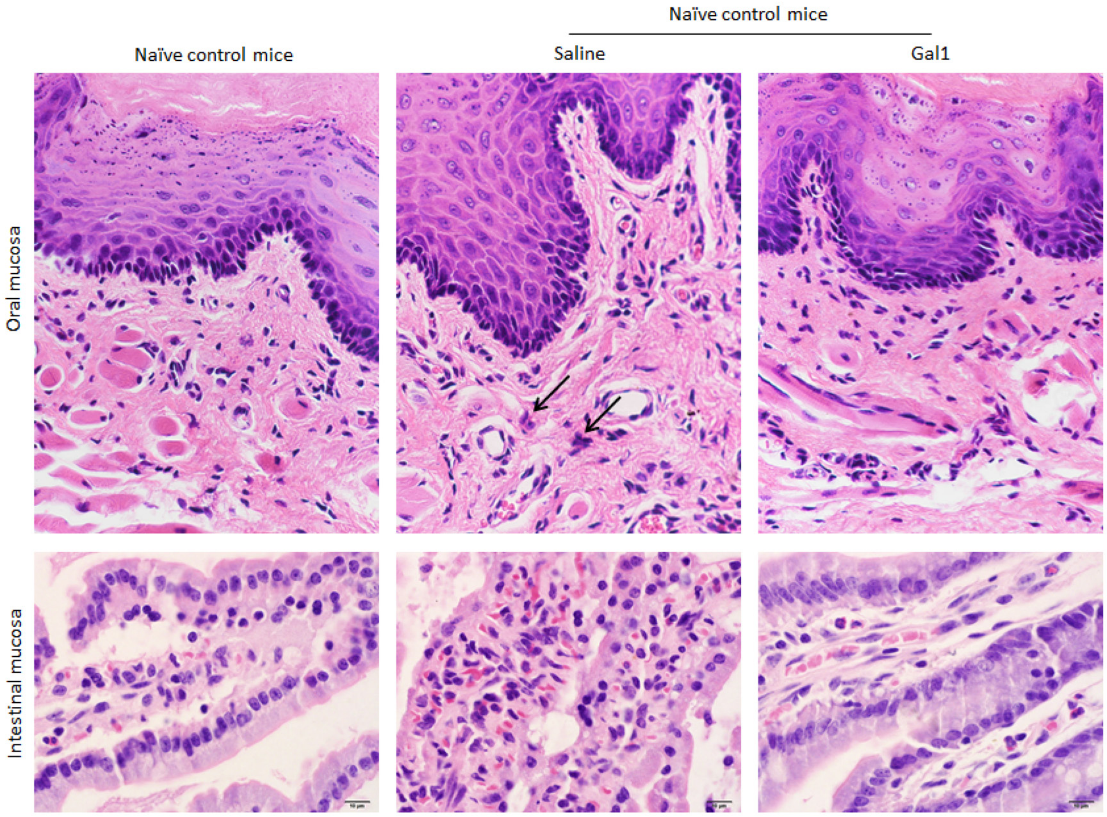

Supplementary Figure 1: Eosinophils in the oral mucosa and intestinal mucosa. Supplemental to Fig. 1C. Eosinophils were those stained in pink cytoplasm (pointed by arrows). Magnification: ×400.

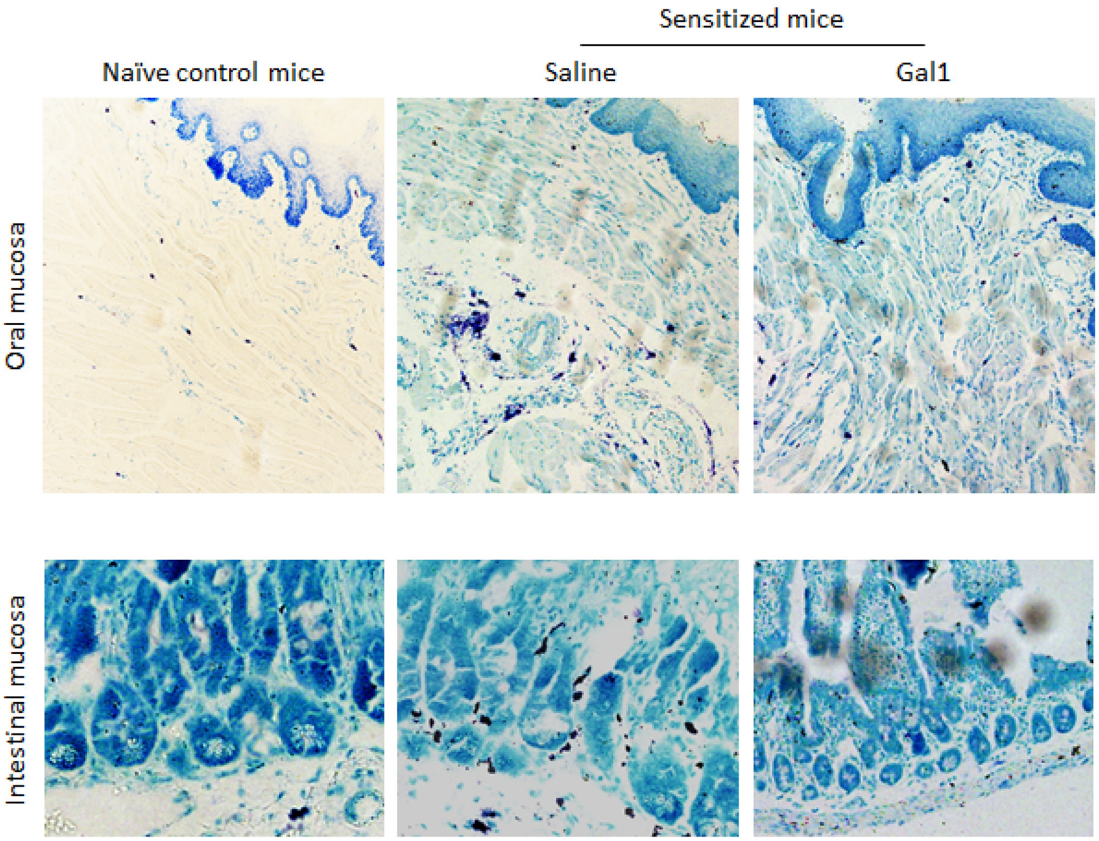

Supplementary Figure 2: Mast cells in the oral mucosa and intestinal mucosa. Supplemental to Fig. 1D. Mast cells were stained in dark blue. Magnification: ×200.
